# Fragile X-Associated Tremor/Ataxia Syndrome (FXTAS): Pathophysiology and Clinical Implications

**DOI:** 10.3390/ijms21124391

**Published:** 2020-06-20

**Authors:** Ana Maria Cabal-Herrera, Nattaporn Tassanakijpanich, Maria Jimena Salcedo-Arellano, Randi J. Hagerman

**Affiliations:** 1 UC Davis MIND Institute, UC Davis Health, Sacramento, CA 95817, USA; ana.cabal@correounivalle.edu.co (A.M.C.-H.); tnattaporn.ped@gmail.com (N.T.); mjsalcedo@ucdavis.edu (M.J.S.-A.); 2 Group on Congenital Malformations and Dysmorphology, Faculty of Health, Universidad del Valle, Cali, Valle del Cauca 760041, Colombia; 3 Department of Pediatrics, Faculty of Medicine, Prince of Songkla University, Songkhla 90110, Thailand; 4 Department of Pediatrics, University of California, Davis, School of Medicine, Sacramento, CA 95817, USA

**Keywords:** fragile X-associated tremor/ataxia syndrome (FXTAS), *FMR1*, premutation, FMRP, ataxia, tremor, neuroradiology, neurodegeneration

## Abstract

The fragile X-associated tremor/ataxia syndrome (FXTAS) is a neurodegenerative disorder seen in older premutation (55–200 CGG repeats) carriers of *FMR1.* The premutation has excessive levels of *FMR1* mRNA that lead to toxicity and mitochondrial dysfunction. The clinical features usually begin in the 60 s with an action or intention tremor followed by cerebellar ataxia, although 20% have only ataxia. MRI features include brain atrophy and white matter disease, especially in the middle cerebellar peduncles, periventricular areas, and splenium of the corpus callosum. Neurocognitive problems include memory and executive function deficits, although 50% of males can develop dementia. Females can be less affected by FXTAS because of a second X chromosome that does not carry the premutation. Approximately 40% of males and 16% of female carriers develop FXTAS. Since the premutation can occur in less than 1 in 200 women and 1 in 400 men, the FXTAS diagnosis should be considered in patients that present with tremor, ataxia, parkinsonian symptoms, neuropathy, and psychiatric problems. If a family history of a fragile X mutation is known, then *FMR1* DNA testing is essential in patients with these symptoms.

## 1. Fragile X Syndrome and Associated Disorders

Fragile X syndrome (FXS) is the most common cause of inherited intellectual disability and autism spectrum disorder (ASD). It results from the expansion of the CGG trinucleotide repeats (>200) in the promoter region of the fragile X mental retardation 1 (*FMR1*) gene, located at Xq27.3 [1]. The expansion leads to methylation and subsequent silencing of the gene. In some rare cases, less than 1%, FXS occurs due to other defects that lead to loss of function of the gene, such as deletions or point mutations [2,3]. The *FMR1* gene normally codes for the fragile X mental retardation protein (FMRP) and therefore silencing of the gene leads to loss of expression of the protein. FMRP is an RNA binding protein that is involved in several processes including neuronal plasticity and functioning of neuronal networks [4,5,6]. Healthy individuals have less than 45 CGG repeats. Expansion to 46–54 repeats is considered the gray zone and individuals with 55–200 repeats are considered to have the premutation (PM) [7]. Carriers of the PM can present with conditions such as fragile X-associated primary ovarian insufficiency (FXPOI) in females [8], neuropsychiatric conditions such as anxiety and depression, recently recognized as fragile X-associated neuropsychiatric disorders (FXAND) [9] and fragile X-associated tremor/ataxia syndrome (FXTAS) [10]. In this review we will focus on FXTAS, a movement disorder characterized by tremor and/or ataxia, cognitive involvement, neuropathy, and autonomic dysfunction in individuals with the PM.

FXTAS was first described in 2001 by Hagerman and colleagues [11] when they reported five elderly males who were carriers of the PM and presented with a progressive intention tremor, difficulty with ambulation, deficits in executive function and brain atrophy in association with elevated *FMR1* messenger RNA (mRNA) levels. Since then, FXTAS has become a well-known progressive neurodegenerative disorder that occurs in PM carriers, both men and women who are aging and present clinically with the core features of intention tremor and/or ataxia [12]. There have been reports of individuals with CGG repeat numbers in the gray zone range presenting with clinical and neuroradiological findings consistent with FXTAS [13,14]. Although rare, there have also been reports of individuals with the full mutation (FM) who present with a clinical picture consistent with FXTAS. These individuals with the FM present with size mosaicism and/or lack of methylation of the *FMR1* gene and therefore have elevated mRNA levels and some production of FMRP [15,16,17]. These cases have made experts consider modifications of clinical criteria for diagnosing FXTAS to not only involve premutation carriers but also those with a gray zone or FM repeat number [18].

It is important for physicians to be aware of the PM and its associated disorders since it is likely they will encounter a patient with the PM in their clinic, and if they are unaware of their carrier status, they can misdiagnose conditions such as FXPOI or FXAND in addition to FXTAS. A meta-analysis conducted by Hunter and colleagues in 2014 determined that the prevalence of the PM in the general population is approximately 1 in 300 females and 1 in 850 males [19]. It is noteworthy that the prevalence varies greatly globally with some countries reporting regions with prevalence as high as 1 in 28 females and 1 in 71 males [20] and with Japan being the country with the lowest reported prevalence of the PM allele [21].

## 2. Epidemiology of FXTAS

The only prevalence study of FXTAS among the premutation population was a total ascertainment study of all known fragile X families in California [22]. They found that age was associated with an increased prevalence of tremor and ataxia in male carriers such that those in their 50s had a prevalence of 17% but those in their 80s had a prevalence of 75%. However, there are no prevalence studies in the general population [12]. Many studies have addressed the premutation prevalence in patients presenting with movement disorders [23] such as screening studies in adult patients presenting with ataxia [24,25], spinocerebellar ataxia [26], multiple system atrophy [27,28,29], parkinsonism [30,31,32] and essential tremor [33,34,35]. Overall, the prevalence of *FMR1* expansions in populations being evaluated for movement disorders is low (<2%) but this could be explained by several factors such as lack of insight of patients with FXTAS into their symptoms resulting in them not reporting their neurological symptoms [36] and the fact that few patients with FXTAS are being referred to and evaluated by a movement disorder specialist [37]. Most of these studies concluded that due to the low prevalence of *FMR1* expansions in the populations studied, genetic testing should only be done if there are additional clinical features of fragile X- associated disorders or family history of fragile X disorders.

FXTAS is an age-dependent neurodegenerative disorder with prevalence increasing with age and affecting most commonly males [38]. It is estimated that approximately over 40% of males with the PM will eventually present FXTAS [22] in contrast with female carriers in whom it is estimated that approximately 13% to 16% of them will be affected by FXTAS [39]. Other studies have shown that the penetrance may be also related to the CGG repeat size with lower repeats associated with lower penetrance [22,40,41]. In the largest retrospective study of FXTAS to date, 55 men were included and the average age of symptom onset was 60.6 years (+/−8.6 years) [42]. There are some reports of patients presenting with early onset of FXTAS in which they have had an environmental exposure that may have contributed to the earlier presentation and increased severity of symptoms; such as exposure to neurotoxins [43], pollution, illicit drugs, pesticides, and alcohol consumption [44,45,46]. The progression of FXTAS varies greatly among individuals and the life expectancy after symptoms onset has been established to be between 5 to 25 years with a median of 21 years [42].

## 3. Clinical Presentation of FXTAS

**Figure 1:**
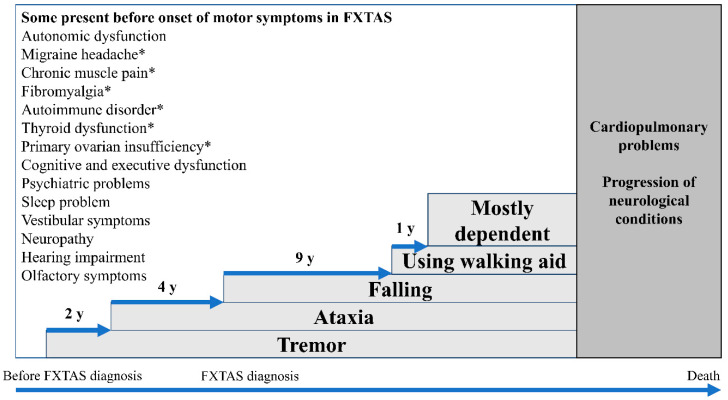
Clinical manifestations of the fragile X premutation and fragile X-associated tremor/ataxia syndrome (FXTAS) overtime. * Clinical manifestations which are more prevalent in females with the fragile X premutation than males. (Adapted from Leehey et al. 2007; Greco et al. 2006) [42,75].

The core presenting features of FXTAS are intention tremor and cerebellar ataxia. These usually present at a mean age of 62 with an action or intention tremor followed by the development of an ataxic gait; although 20% of patients have only ataxia [18]. Neuropathy, parkinsonism, and executive dysfunction are commonly associated with FXTAS [47]. In addition, several conditions are prevalent in PM carriers with and without FXTAS which include dysautonomia [18,48,49,50,51], sleep problems [52,53], migraine headaches [54], vestibular dysfunction [55], hearing deficit [56], olfactory deficit [57], chronic fatigue [58], and psychiatric problems [9,59,60,61,62]. Fibromyalgia [51,63], autoimmune disorders [64], and thyroid dysfunction [51] are common in females with FXTAS. Figure 1 illustrates the typical clinical progression of FXTAS.

Clinical presentation, age of onset, and severity of FXTAS are heterogeneous. A larger CGG repeat size in PM carriers predicts earlier age of onset and perhaps earlier death [40,41,65]. Symptoms in females are milder since they have two X chromosomes [39,66]. The first five females with FXTAS reported did not have dementia [66] but subsequent reports have included female cases of FXTAS with dementia [66,67]. Increasing X-inactivation ratio and skewed inactivation of the mutated allele in females can be a protective factor [66,68,69]. Lower AGG interruptions and elevated expression of *antisense FMR1* transcript/splice variant 2 (*ASFMR1*-TV2) might predict the development of FXTAS [70]. Moreover, the onset and progression of FXTAS might be accelerated if the PM carriers have a history of exposure to toxic substances. A dramatic decline in neurocognitive and neuromotor function after unrelated-FXTAS surgery requiring general anesthesia [43,71], chemotherapy for breast cancer [72], exposure to chemical agents including insecticides, pesticides, herbicides, and chemicals for building [43], and chronic substance use [45,73,74] have been reported.

### 3.1. Tremor

The peak age of onset of tremor in males with the PM is usually in their early 60s [41,42], approximately 2 years prior to the onset of ataxia [42]. The tremor is typically an action or intention tremor but may also be present with holding a position [56,76]. Almost half of the patients do not notice their tremor although it can be seen with writing, drinking, and eating [18]. Head tremor, titubation, and voice tremor are found in 10% of patients with FXTAS [56]. Resting tremor occurs in 13–26% of individuals with FXTAS and it typically co-occurs with other types of tremor [56,76,77].

### 3.2. Cerebellar Gait Ataxia

The average age of onset of ataxia is 63.6 ± 7.3 years [41]. Cognitive function is also often impaired when ataxia is detected [56]. Difficulty in performing tandem gait, taking a longer time to turn, and increased gait variability are the initial signs of ataxia [56,78]. These lead to gait instability and falling [18,42]. Dysmetria and dysarthria are commonly noted on examination [18].

### 3.3. Neuropathy

Around 80% of individuals with FXTAS have peripheral neuropathy, although it is underrecognized by patients [76]. Neuropathy usually presents before motor symptoms of FXTAS develop [12] and it can be the presenting symptom of FXTAS [79]. Numbness and neuropathic pain are typical; impaired vibration sense and abnormal deep tendon reflexes are usually detected [76,79,80,81,82]. A study in 16 individuals with FXTAS found that sensory axonal neuropathy was the most predominant characteristic of neuropathy in FXTAS with reduction of the sensory nerve action potential amplitude demonstrated in nerve conduction studies [76]. All extremities could be involved and both non-length-dependent and length-dependent sensory neuropathy were described [76]. In addition, motor neuropathy has also been observed [76,79,83]. In male carriers, the severity of neuropathic signs and degree of changes in nerve conduction studies correlated with the number of CGG repeats, mRNA level, and having ataxia [81,83]. Intranuclear inclusions in the dorsal root ganglia might underlie neurodegeneration which causes the neuropathy [48,84].

### 3.4. Parkinsonism

Parkinsonism has been found in approximately 29–60% of carriers with FXTAS and it is usually mild [18,56,76,77]. A variety of parkinsonian features have been observed in FXTAS. Mild bradykinesia, rigidity of the upper extremities while performing mirror movements, masked facies, and rest tremor have been reported. Bradykinesia is correlated with the level of *FMR1* mRNA, ataxia, and the stage of FXTAS [18,56,77]. The presence of bradykinesia with cerebellar gait ataxia and mixed tremor should alert physicians to recognize FXTAS in the differential diagnosis of Parkinson’s disease [77].

### 3.5. Eye Gaze Abnormalities

Eye gaze abnormalities have been described in patients with FXTAS, especially impaired optokinetic nystagmus in the vertical direction, slowing of vertical saccades, saccadic pursuits, and square wave jerks [82,85]. These findings are referred to as the progressive supranuclear palsy (PSP)-like phenotype of FXTAS. Brainstem atrophy particularly in the midbrain region has been found in both FXTAS and PSP, although it is unclear whether they are co-occurrences or whether FXTAS promotes PSP changes in the phenotype [82]. Further pathological studies will clarify the primary mechanism.

### 3.6. Cognitive Impairment

Approximately half of males with FXTAS have cognitive impairment while females are less affected [56,60,86,87]. Executive dysfunction is the core cognitive deficit in FXTAS. Behavioral self-regulation, verbal fluency, working memory, remote recall, declarative verbal learning memory, visuospatial function, temporal sequencing, information processing speed, and general intelligence, specifically nonverbal intellectual quotient, are often found to be impaired in individuals with FXTAS [88,89,90]. The impairment is correlated with CGG repeat number [65,88,89] and it worsens over time. Milder cognitive impairment can sometimes affect non-FXTAS carriers who are also aging [90]. Cognitive decline sometimes precedes motor problems, and carriers with mid-range CGG repeats have the highest relative risk compared to carriers with lower or higher CGG repeats [86,91].

Unlike dementia in Alzheimer’s disease which usually affects memory and language performance; executive dysfunction and verbal dysfluency which reflect cortical-subcortical dementia are distinct in FXTAS [87]. In addition, FXTAS has a higher penetrance in males and should be suspected in patients presenting with dementia and atypical parkinsonian syndrome [87]. Moreover, compared to mothers of children with ASD who were considered to have similar parental experiences to the PM mothers of children with FXS, the latter group had higher scores in verbal dysfluency and impaired self-monitoring [92]. Furthermore, in a study by Nayar and colleagues, they examined language processing skills in 46 females with the PM and 56 controls and they found a pattern of inefficient language processing among the females with the PM [93]. In individuals with FXTAS, it is necessary to address cognitive impairment and implement early intervention since impaired information processing speed, response inhibition, and working memory could interfere with their mobility [94].

### 3.7. Other Coexisting Conditions

Autonomic dysfunction is common in males with FXTAS. Impotence (56–80%), hypertension (50–67%), and orthostatic hypotension (16%) usually present before the onset of motor symptoms. Bowel (30%) and bladder dysfunction (24–55%) occur at later stages of FXTAS [18,50,56]. In both sexes, individuals with FXTAS have higher odds of having hypertension compared with age-matched noncarriers and the risk of having hypertension is related to *FMR1* mRNA levels [47,50,51]. Cardiac arrhythmia is not uncommon and several reports have consistently described individuals with FXTAS who presented with arrhythmias that required a cardiac pacemaker [43,67,75]. Recently sudden coronary artery dissection (SCAD) has been reported in three older females with the premutation [95]. Rare symptoms of autonomic dysregulation have been reported in individuals with FXTAS including postprandial hypotension [96] and postprandial syncope [97].

Sleep problems, especially obstructive sleep apnea (OSA), are diagnosed in about one-third of the carriers with FXTAS [52,56]. One-third of the PM carriers also have restless legs syndrome [53].

Neuropsychiatric problems are quite common in carriers and usually present early in life. Anxiety and depression are found in half of the PM carriers and the prevalence is higher in FXTAS [61,62]. Executive dysfunction, imbalance of GABA and glutamate functions, chronic medical conditions, and caring for children with FXS might be potential risk factors for these neuropsychiatric problems [9]. However once FXTAS begins, often depression, apathy, irritability, and social avoidance are common and usually worsen with cognitive decline [98].

Some clinical characteristics are more prevalent in females with the premutation compared to males. Half of the females with the premutation have a history of migraine headaches in contrast with only one-fourth of male carriers [54]. Thyroid dysfunction, specifically hypothyroidism, is found in 50% of females with FXTAS [64]. More than 25% of females with FXTAS are reported to have fibromyalgia [51,63,64]. These symptoms are usually problematic even in mid-adulthood and they commonly require medical treatment.

## 4. Neuroradiological Findings

Radiological findings, which are discussed here, were inferred from studies in males with FXTAS. Bilateral hyperintensities of the middle cerebellar peduncles (MCP sign) on T2-weighted MR or FLAIR images is the hallmark of FXTAS [18]. The sign can even be seen in some carriers without remarkable neurological symptoms [99,100,101,102]. Hyperintensities are also commonly observed in the periventricular white matter, the splenium of the corpus callosum, and the brainstem [76,103], although these signs are not specific to FXTAS. The MCP sign is found in 58–82% of males and 13% of females with FXTAS [103,104] while the splenium hyperintensity is observed in approximately 60% of the patients, equivalently in both sexes [103].

Brain atrophy and general brain volume loss have been described in several studies. Atrophy can be found in the entire cerebrum and cerebellum, particularly in the dorsomedial frontal-parietal regions, insula, medial temporal regions, thalamus, and striatum [18,105,106,107]. Ventricular enlargement is also present in the later stages of FXTAS as well as thinning of the corpus callosum [105,108,109]. In females with FXTAS, the magnitude of cerebellar volume loss and the severity of white matter disease is less than in males [104].

The number of CGG repeats associates with the degree of structural brain changes. Increasing CGG repeat numbers correlate with a greater degree of cerebral and cerebellar atrophy in male carriers [104], volume loss in the supplementary motor area and the dorsomedial prefrontal cortex [106], ventricular enlargement, and whole-brain white matter hyperintensity and volume [108,109,110]. In addition, diffusion tensor imaging (DTI) studies revealed abnormalities of the white matter fiber tracts of the middle and superior cerebellar peduncles, and these findings are correlated with CGG repeat number and *FMR1* mRNA level [111,112].

These radiological abnormalities which are in various brain regions correlate to clinical manifestations of FXTAS [113]. For instance, motor control and dexterity relate to the abnormal findings in the corpus callosum and superior cerebellar peduncle [114]. Cerebellar atrophy might result in postural instability [115]. Widespread white matter hyperintensities and grey matter loss as well as decreased prefrontal cortex activity are correlated with compromised cognitive and executive functions [106,108,116,117]. Reduced hippocampal volume and left amygdala grey matter loss is associated with increasing of psychiatric symptoms [106,115,118].

## 5. Pathophysiology

### 5.1. Molecular Mechanisms

The neurotoxic effect of increased levels of *FMR1* mRNA has been proposed as the main alteration leading to the development of FXTAS [12]; increased levels of mRNA also cause Ca^+2^ dysregulation followed by mitochondrial dysfunction [119,120]. Three different molecular mechanisms have been studied with the aim of understanding the molecular abnormalities resulting in the neuropathology of FXTAS: (1) the production of toxic FMRpolyG by repeat associated non-AUG (RAN) translation, (2) RNAs and protein sequestration into intranuclear inclusions and (3) DNA damage caused by R-loop formation.

**Figure 2:**
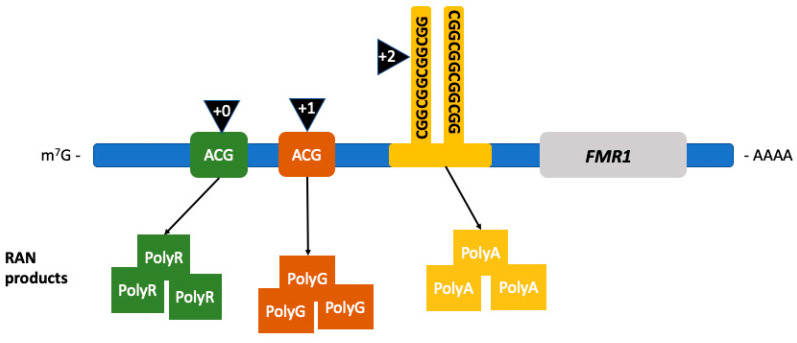
Repeat associated non-AUG (RAN) translation in FXTAS. RAN translation leads to the production of RAN products depending on the location where the initiation of translation occurs. Initiation (black triangles) occurring in the +0 reading frame leads to the production of FMRpolyR (green squares), +1 to FMRpolyG (brown squares), and +2, within the CGG-repeat region, to the production of FMRpolyA (yellow squares). (Adapted from Glineburg et al. 2018) [127].

The first proposed mechanism is RAN translation [121]. RAN translation is common in triplet expansion disorders. This error in translation leads to anomalous peptide synthesis [121,122,123,124,125]. Particularly in FXTAS, the noncoding region of *FMR1* premutation mRNA is translated into multiple RAN translation peptides, as seen in Figure 2, including the FMRpolyG peptide [121,126]. This peptide has been found to be toxic by disrupting the architecture of the nuclear lamina due to its interaction with the transmembrane protein lamina-associated polypeptide 2 beta (LAP2β) in differentiated neurons derived from FXTAS induced pluripotent stem (iPS) cells. The length of FMRpolyG correlates with the number of CGG repeats; it is detected in CGG expansions ranging from 60 to 200 repeats and it is also present in the intranuclear inclusions pathognomonic of FXTAS in the *FMR1* premutation transgenic animal models [126].

**Figure 3:**
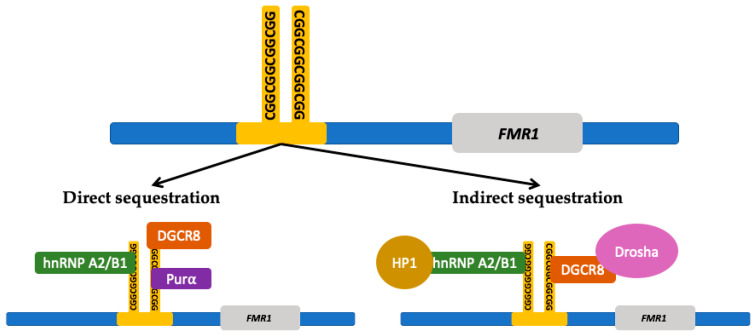
*FMR1* RNA induced protein sequestration. The expanded CGG-repeat region in the *FMR1* free mRNA can form higher-order structures that can sequester proteins which results in a deficiency within the cell from those proteins. The CGG-repeat region is directly bound by repeat binding proteins such as heterogeneous nuclear ribonucleoproteins A2/B1 (hnRNP A2/B1), DiGeorge syndrome critical region 8 (DGCR8) protein, and Pur-alpha ((Purα) protein. The CGG-repeat region can also indirectly sequester other proteins through the interaction with the directly bound proteins. Proteins that can be indirectly sequestered include Drosha and heterochromatin protein 1 (HP1). (Adapted from Hagerman and Hagerman 2016 and Glineburg et al. 2018) [12,127].

The second mechanism is the sequestration of RNA and other proteins by the *FMR1* premutation mRNA forming intranuclear inclusions, disrupting essential cellular processes. Protein sequestration renders the cell functionally deficient from proteins that play an important role in multiple cellular mechanisms such as splicing (heterogeneous nuclear ribonucleoproteins A2/B1 protein (hnRNP A2/B1)), mRNA transportation in the cytoplasm (hnRNP A2/B1, Pur-alpha), microRNA processing (DiGeorge Syndrome Critical Region 8 (DGCR8) protein and Drosha complex), and formation of heterochromatin (heterochromatin protein 1 (HP1)) [127,128]. Figure 3 shows the proteins involved in *FMR1* RNA-induced sequestration. Ubiquitin positive inclusions are the hallmark of FXTAS [75,129,130]; they contain *FMR1* mRNA but lack FMRP [131,132]. Inclusions are an aggregate of protein, composed of a heterogeneous assortment of many peptides. The DNA damage response proteins found in the inclusions form in response to oxidative stress leading to neurodegeneration [132]. The mechanisms by which protein sequestration leads to toxicity and disease are currently being studied.

The third proposed molecular mechanism involves the formation of RNA/DNA hybrids or R-loops during transcription. R-loops form during the CGG triplet expansion at the *FMR1* locus causing vulnerability to DNA damage [133]. These R-loops are more frequently formed in the nontemplate strand containing guanine-rich sequences [134,135]. These folded structures remain unpaired and lead to genome instability [136]. The sites become fragile and are prone to DNA damage including deletions and translocations. DNA damage should be corrected by the DNA damage response (DDR) molecular signaling pathway [137]. However, this response appears to be impaired in FXTAS [138].

The molecular mechanisms presented here do not exclude one another; on the contrary, there may exist a synergistic effect from different dysfunctional processes at the molecular level leading to FXTAS. The mitochondrial abnormalities present in FXTAS may worsen over time and lead to loss of energy and strength in the patients and perhaps neuronal cell death and white matter disease over time [119,139,140,141]. Many additional mechanisms causing neuropathology and toxicity may remain unrecognized.

### 5.2. Pathology

One of the neuropathological hallmarks of FXTAS is the presence of eosinophilic intranuclear inclusions in the CNS and peripheral nervous system [22,84]. The presence of inclusions in neurons and astrocytes constitutes a major criterion for the diagnosis of FXTAS [22,142]. These intranuclear inclusions were first identified in neurons and astrocytes in the brain in 2002 by Greco and colleagues [129]. Inclusions occur broadly throughout the CNS with regional variability in the proportion of cells bearing the inclusions [143]. Neurons and astrocytes positive for the presence of inclusions are mostly prevalent in the hippocampus (up to 40% of cells), followed by the frontal and the temporal cortex [67,75,129]. These inclusions have also been identified in the basal ganglia [97]. Further studies identified these inclusions in the peripheral nervous system, mainly in the autonomic system [48], and other non-CNS locations such as neuroendocrine tissue, heart, and kidney [84,97,144].

Various studies have been conducted in order to elucidate the features of the inclusions. These eosinophilic inclusions have shown to stain positive for ubiquitin, lamin A/C and various heat-shock proteins, and their biochemical composition is very heterogeneous with no single predominant protein species [131]. Mass spectrometric and immunohistochemical analysis in postmortem FXTAS brain tissue have identified more than 20 inclusion-associated proteins including RNA-binding protein, hnRNP A2, intermediate filament proteins, and other neurofilament proteins [75,129,131]. *FMR1* mRNA was also identified but as a minor component [145]. More recently, Ma and colleagues conducted a study of the composition of the intranuclear inclusions of FXTAS using fluorescence-activated cell sorting (FACS) and liquid chromatography/tandem mass spectrometry-based proteomics. They identified more than 200 proteins which are normally involved in RNA binding, protein turnover, and DNA damage repair [132]. It is noteworthy that the presence of intranuclear inclusions has also been identified in a carrier with no clinical symptoms associated with FXTAS, although this carrier was bedridden with severe ataxia and perhaps other symptoms associated with FXTAS by the time she died, so it is uncertain if the presence of inclusions always means FXTAS, but this is likely the case [67].

Iron metabolic pathway dysregulation has been identified as a mechanism underlying the neuropathology of FXTAS and therefore as a potential targeted treatment. Postmortem FXTAS brains present with iron accumulation in brain capillaries and parenchyma, as well as in the choroid plexus [146,147,148]. Severe iron accumulation is a common finding in the striatum and less commonly in the cerebrum [146]. There is a decreased amount of transferrin and ceruloplasmin, which are iron-binding proteins, in neurons and astrocytes, whereas there are increased levels of these proteins in the microglial cells, which indicates an attempt to respond to excessive iron accumulation [146]. Studies have demonstrated substantial iron bound to hemosiderin accumulated in the putamen [146,147,149], which on neuroimaging can be detected as symmetric hypointensities in the putamen and caudate in T2-weighted MRI [85,114].

Since FXTAS is associated with high levels of oxidative stress in the brain and an inflammatory state, and considering that microglia-mediated neuroinflammation plays a major role in the pathogenesis of neurodegenerative diseases, it was proposed that microglial activation could contribute to FXTAS pathology [150]. In a study of 13 FXTAS brain specimens, Martínez-Cerdeño and colleagues found that almost half of FXTAS brains presented with dystrophic senescent microglial cells, suggesting that these could be used in association with the presence of intranuclear inclusions and iron deposits as a marker in the postmortem diagnosis of FXTAS [150]. They also found that the presence of senescent microglia was correlated with the CGG repeat number and with iron accumulation. This suggests that microglial cells are involved in the neuroinflammatory state of FXTAS and that they can serve as a pathological criterion for the diagnosis.

FXTAS can coexist with other neurodegenerative diseases. Salcedo-Arellano and colleagues conducted a study to describe the frequency of Parkinson’s disease and concomitant FXTAS [151]. They reviewed medical records and performed a pathology analysis on 40 deceased patients with a diagnosis of FXTAS. They found that 5% of the brains of 40 patients met pathologic criteria for both Parkinson’s disease, based on the presence of Lewy bodies in the substantia nigra and nigral neuronal loss, as well as FXTAS. In the case of Alzheimer’s disease, some patients with FXTAS and dementia symptomology presented with concurrent findings of Alzheimer’s neuropathology such as amyloid plaques and neurofibrillary tangles [67]. Various case reports of patients with FXTAS and coexisting Alzheimer’s disease or Parkinson’s disease suggest a synergistic effect on the progression of FXTAS and the concomitant neurodegenerative disease [67,152,153].

Finally, macroscopic pathological findings of brains from patients with FXTAS reveal severe white matter disease, cortical atrophy and mild to severe ventriculomegaly and spongiosis of cerebellar white matter [67,75,129]. Grey matter atrophy is most consistently found in the frontal cortex, cerebellum, and the pons [75,129].

## 6. Diagnosis

### 6.1. Diagnostic Criteria for FXTAS

**Figure 4:**
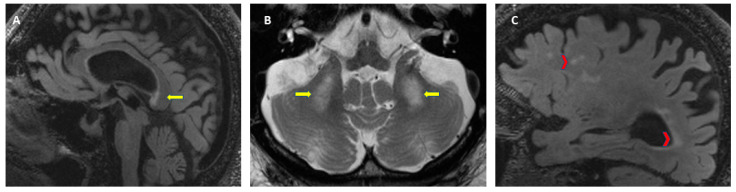
Neuroradiological criteria for the diagnosis of FXTAS. (**A**) T2-FLAIR: white matter lesions in the splenium of the corpus callosum, (**B**) T2-TSE: symmetrical white matter lesions in the middle cerebellar peduncles (MCP sign), (**C**) T2-FLAIR: cerebral white matter lesions and brain atrophy.

As described earlier, FXTAS is characterized by multiple clinical signs, although there is heterogeneity in the severity depending on the sex of the patient, CGG repeat size, and length of disease. For the diagnosis of FXTAS a molecular diagnosis of an *FMR1* gene mutation, including the gray zone, is needed. It is important to know the CGG repeat size since it correlates negatively with the age of onset of symptoms [41] and it correlates positively with the severity of motor signs and brain atrophy [40,154]. The diagnostic criteria for FXTAS were established for the first time in 2003 [18] and later revised in 2014 to expand the radiological and clinical criteria [155]. Due to reports of FXTAS in patients with the gray zone and FM with a lack of methylation or mosaic alleles, the molecular criteria is now considered to be any *FMR1* alteration [13,14]. See Table 1 for FXTAS diagnostic criteria and diagnostic categories. When FXTAS is suspected, the clinician needs to perform molecular, clinical, and neuroradiological evaluations. The *FMR1* gene testing characterizes the *FMR1* mutation, including the gray zone (45 to 54 CGG repeats), which is the only required criteria. A brain MRI allows the evaluation of white matter lesions and brain atrophy; with the presence of white matter lesions in the MCP or brainstem constituting a major radiological sign. These radiological criteria are evaluated on axial flair T2-weighted magnetic resonance images. Figure 4 shows the neuroradiological findings that are part of the diagnostic criteria for FXTAS. A thorough clinical evaluation to assess for intention tremor, cerebellar ataxia, parkinsonism, neuropathy, memory impairment, and executive function deficits is essential for the diagnosis.

When should a physician consider genetic testing for *FMR1* and a FXTAS diagnosis? There are guidelines indicating when and whom to test for *FMR1* gene mutations [156]. Expansions in *FMR1* are diagnosed in relatives as part of cascade testing when a child is diagnosed with FXS or when a woman in the context of fertility testing is diagnosed with the PM and FXPOI [12]. When a clinician encounters a patient with any of the various clinical signs consistent with FXTAS, they should perform a thorough family history and ask further about *FMR1*-related conditions. Questions should include features of FXS such as developmental delay, intellectual disability, and autism spectrum disorders. Questions related to PM carrier status include the age of menopause and features of menopause before the age of 40 for FXPOI, psychiatric symptoms such as anxiety, and movement disorders. A clinician evaluating a patient with a new onset of ataxia or intention tremor and a history of a grandchild with autism or developmental delay of unknown etiology should consider FXTAS among the differential diagnosis.

### 6.2. Differential Diagnosis

When evaluating for FXTAS, the clinician must consider and rule out other causes of dementia, especially reversible causes. The differential diagnosis of FXTAS is broad and some neurodegenerative diseases may coexist. Among the differential diagnosis of FXTAS are movement disorders and dementias such as late-onset cerebellar ataxia, spinocerebellar ataxia, multiple system atrophy, Alzheimer’s disease, frontotemporal dementia, and Parkinson’s disease. Robertson and colleagues reviewed the cognitive and motor impairments in FXTAS and compared it with other movement disorders that patients with FXTAS are sometimes misdiagnosed with [157]. Since there may be symptomatology overlap, it is important to know the most common presentations and the distinct profiles in cognitive and motor domains to guide the diagnostic process.

## 7. Management and Ongoing Research

FXTAS is a neurodegenerative disorder caused by RNA toxicity and mitochondrial dysfunction related to *FMR1* gene mutations. Principles of management include maintaining a healthy lifestyle and treatment of coexisting conditions to prevent rapid progression of FXTAS. Hypertension, OSA, hypothyroidism, neuropsychiatric disorders, and vitamin deficiencies can potentiate cognitive decline. These conditions need to be evaluated and treated in all PM carriers [158]. To date, there are not any targeted treatments that will reverse FXTAS, but some medications may help symptoms of FXTAS such as a selective serotonin reuptake inhibitor (SSRI) for depression or anxiety, a beta blocker, primidone or levetiracetam for tremor, and gabapentin, pregabalin or duloxetine for neuropathic pain [12]. Many studies have investigated benefits of medications that have been used for other neurodegenerative disorders. We will mention the drugs that have been studied in clinical trials in patients with FXTAS and have published results to date.

Memantine is a medicament that blocks the *N*-methyl-d-aspartate (NMDA) receptor which is a subtype of glutamate receptor [159]. It has been approved for the treatment of Alzheimer’s disease. A randomized controlled trial was conducted in 94 individuals with FXTAS in which memantine titrated to 10 mg was prescribed to 43 participants. After one year follow-up, the memantine group had a comparable degree of tremor, executive function score, and verbal fluency score compared to the control group [160]. An inadequate sample size and few participants with later stages of FXTAS might have compromised the power of the study. However, subsequent studies in the same participants found benefits of memantine on verbal memory, attention, and working memory which were evaluated by using event-related potential (ERP) [161,162]. Therefore, memantine appears to be helpful for auditory processing involved in attention and aspects of cognition including memory, and it can be tried in patients with FXTAS.

Allopregnanolone is a neurosteroid that is synthesized from progesterone. It is a potent positive allosteric modulator and acts on GABA_A_ receptor. Therefore, allopregnanolone might promote neurogenesis and has neuroprotective effects. An open-label trial of weekly titrated 2 to 6 mg of intravenous allopregnanolone for 12 weeks was studied in six males aged 57–74 years diagnosed with the PM and FXTAS stages 3–5 [163,164]. Improvement of scores in executive function, memory, and learning was noted [163,164], especially in the participants with preserved hippocampus and corpus callosum sizes on MRI [164]. Semantic processing and verbal learning memory, which was measured by ERP N400 word repetition effect while performing a semantic memory processing task, was also improved [164]. One patient had a dramatic improvement in neuropathy symptoms. Decreased anxiety symptoms were also observed in the participants with small hippocampus and amygdala [164]. Allopregnanolone treatment significantly improved GABA metabolism and metabolites of mitochondrial function and reduced oxidative stress. It also increased N-acetylornithine which might improve motor control [163].

Citicoline, cytidine-5-diphosphocholine, is essential for cell membrane stabilization and inhibition of free radicals. It has been used for the treatment of head trauma, ischemic cerebrovascular disease, and neurodegenerative disease [165]. Recently, an open-label phase II pilot study of citicoline was conducted in ten participants aged 70 ± 7.3 years old with CGG 91.5 ± 15.6 repeats and FXTAS stage 1–3 [166]. Citicoline in a dose of 1000 mg was prescribed once daily for 12 months. Stable motor functions were observed after 1-year citicoline, evidenced by no significant change in FXTAS rating scale (FXTAS-RS), which is a tool designed to measure the severity of motor symptoms in FXTAS. However, citicoline had promising results in other secondary outcomes: it showed improvement of anxiety and response inhibition without causing serious adverse events. Control clinical trials are needed to confirm the findings.

**Figure 5:**
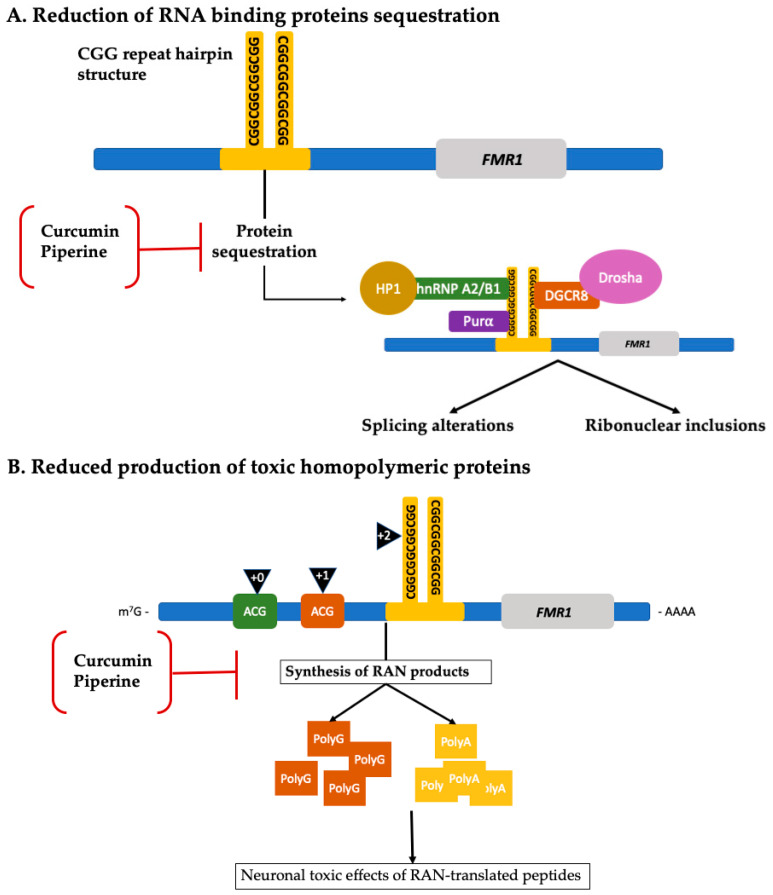
New therapeutic approaches and associated molecular pathways. (**A**) Compounds such as curcumin and piperine have shown to selectively bind the CGG repeat hairpin structure, inhibiting sequestration of RNA and other proteins that are normally involved in cellular mechanisms such as splicing, microRNA processing, and RNA transportation. By inhibiting the sequestration of such proteins, the cell is no longer functionally deficient from them, improving splicing alterations and reducing ribonuclear inclusions formation. (**B**) Curcumin and piperine can also decrease the synthesis of toxic homopolymeric proteins such as FMRpolyG (brown squares) and FMRpolyA (yellow squares), therefore reducing neuronal toxic effects caused by them [167,168]. (Adapted from Hagerman and Hagerman 2016; Glineburg et al. 2018) [12,127].

Although to date, only three clinical trials evaluating potential therapeutic targeted treatments have been completed, other compounds such as curcumin and piperine have shown promising preclinical results [167,168]. See Table 2 for details about their mechanism of action. Figure 5 shows the molecular pathways targeted by curcumin and piperine in preclinical studies. There is no cure for FXTAS and none of the drugs mentioned previously provide enough evidence for their clinical use in FXTAS. There is a need for continuing clinical trials in the search for a targeted treatment. Current management of FXTAS focuses on supportive and symptomatic treatment.

## 8. Conclusions

FXTAS is a neurodegenerative disorder that presents mainly as intention tremor and/or ataxia with cognitive decline, neuropathy, and autonomic dysfunction. FXTAS occurs mostly in PM carriers. Given the high prevalence of PM alleles, it is likely that a clinician will encounter a patient who is a PM carrier. It is important to consider FXTAS in the differential diagnosis of adults presenting with movement disorders, especially if there is a family history consistent with FXS or premutation disorders such as FXPOI or FXAND. Clinicians who are unaware of the carrier status of their patients may misdiagnose FXTAS, and it is therefore important to perform a thorough family history and consider genetic testing for *FMR1* repeat expansion when indicated. There are currently no targeted treatments for FXTAS, but there are ongoing clinical trials. It is important for PM carriers to modify their lifestyle and prevent noxious environmental exposure in order to prevent or delay FXTAS symptoms.

## Figures and Tables

**Table 1:** Diagnostic criteria and categories for FXTAS. Adapted from Hall et al. 2014 [155].

Diagnostic Criteria		
Molecular	Required	*FMR1* Mutation *
Clinical	Major	Intention tremor
		Cerebellar gait ataxia
	Minor	Parkinsonism
		Neuropathy
		Memory and executive function deficits
Neuroradiological	Major	White matter lesions in the middle cerebellar peduncles (MCP sign) or brainstem
	Minor	White matter lesions in the splenium of the corpus callosum
		Cerebral white matter lesions
		Moderate–severe brain atrophy
Neuropathological	Major	Ubiquitin-positive intranuclear inclusions
Diagnostic categories		
	Definite	1 clinical major AND 1 neuroradiological major OR 1 neuropathological major
	Probable	2 clinical major OR 1 clinical minor + 1 radiological minor
	Possible	1 clinical major + 1 clinical minor

* FMR1 mutation includes premutation, gray zone and FM with mosaicism.

**Table 2:** New therapeutic approaches and associated molecular pathways.

Medication/Compound	Molecular Mechanism	Clinical Trial Phases Completed	Reference
Curcumin	Decreases RANT and FMRpolyG production, reducing its accumulation.	Preclinical	[167]
	Selectively binds CGG RNA repeats potentially reducing RNA binding proteins sequestration and reducing CGG repeats-induced cell toxicity.		
Cytidine 5′diphospho-choline (Citicoline)	Inhibition of phospholipase A_2_ (PLA_2_) leading to suppression of CGG repeats-induced cell toxicity.	Phase 2	[166,169]
Memantine	Noncompetitive antagonist of N-methyl-d-aspartate (NMDA) proposed to normalize premutation associated abnormal neuronal response to glutamate.	Phase 2	[161,170]
Piperine	Selectively binds CGG RNA repeats potentially reducing RNA binding proteins sequestration and reducing CGG repeats-induced cell toxicity.	Preclinical	[168]
Allopregnanolone	Positive allosteric modulator of GABA receptors.	Phase 2	[163,164,171,172]
	Reduction of caspase-3 protein expression leading to reduced apoptosis.		
	Inhibition of mitochondrial permeability transition pore which is implicated in the intrinsic pathway of apoptosis.		

## Abbreviations

AD	Alzheimer’s disease
*ASFMR1*-TV2	*antisense FMR1* transcript/splice variant 2
DGCR8	DiGeorge Syndrome Critical Region 8
*FM*	Full mutation
*FMR1*	Fragile X Mental Retardation 1 gene
FMRP	Fragile X Mental Retardation Protein
FXAND	Fragile X-associated neuropsychiatric disorders
FXPOI	Fragile X-associated primary ovarian insufficiency
FXTAS	Fragile-X-associated tremor/ataxia syndrome
FXS	Fragile X syndrome
hnRNP A2/B1	heterogeneous nuclear ribonucleoproteins A2/B1
HP1	heterochromatin protein 1
LAP2β	Lamina-associated polypeptide 2 beta
MCP	Middle Cerebellar Peduncles
MRI	Magnetic Resonance Images
PD	Parkinson’s disease
PM	Premutation
PSP	Progressive supranuclear palsy
Purα	Pur-alpha
RANT	Repeat associated non-AUG translation
